# The association between IGF-1 levels and four types of osteoarthritis: a bidirectional and two-step mendelian randomization study

**DOI:** 10.3389/fgene.2024.1366138

**Published:** 2024-07-10

**Authors:** Xiaohan Pan, Minghuang Cheng, Dongxu Li, Zeyu Liu, Qi Yao, Wei Jiang, Xiaojun Zhang, Jie Hao

**Affiliations:** ^1^ Department of Orthopedics, The First Affiliated Hospital of Chongqing Medical University, Chongqing, China; ^2^ Orthopedic Laboratory of Chongqing Medical University, Chongqing, China

**Keywords:** insulin-like growth factor-1, osteoarthritis, body mass index, mendelian randomization (MR), causality

## Abstract

**Background:**

Insulin-like Growth Factor-1 (IGF-1) plays a crucial role in the growth and metabolic functions of various tissues and cells in the body. Recently, there has been increased attention to the association between IGF-1 and osteoarthritis (OA). However, there is controversy in current research regarding the correlation between IGF-1 levels and OA. Furthermore, the specific manner in which Body Mass Index (BMI), a key risk factor for OA, mediates the impact of IGF-1 levels on OA remains unclear.

**Object:**

This study aimed to investigate the bidirectional causal link between IGF-1 levels and OA in four body regions, and to explore how BMI influences the impact of IGF-1 on these types of OA.

**Method:**

Two-sample Mendelian Randomization (MR) and its combined forms were utilized to investigate the bidirectional relationship between IGF-1 levels and four types of OA, as well as the mediating role of BMI in the impact of IGF-1 levels on OA. Data from various Genome-Wide Association Studies (GWAS) and multiple analytical methods, including inverse variance weighted, MR-Egger regression, and weighted median were utilized. Sensitivity analyses, such as MR-Egger intercept, Cochran Q test, leave-one-out, and MR-PRESSO, were conducted to ensure the robustness of the results.

**Results:**

Higher IGF-1 levels are correlated with an increased risk for knee (OR, 1.07; 95% CI, 1.01–1.03; *p* = 1.49e-01; q = 9.86e-03), hip (OR, 1.13; 95% CI, 1.06–1.20; *p* = 7.61e-05; q = 7.44e-05), and hand OA (OR, 1.09; 95% CI, 1.01–1.17; *p* = 1.88e-02; q = 1.15e-02), but not spine OA but not spine OA (OR, 1.05; 95% CI, 0.99–1.10; *p* = 9.20e-02; q = 5.52e-02). Different types of OA do not affect IGF-1 levels. BMI mediates the increase in OA risk associated with higher IGF-1, including indirect spine OA risk through BMI.

**Conclusion:**

The study elucidates the bidirectional causality between IGF-1 levels and OA in various body parts, highlighting BMI’s mediating role in the impact of IGF-1 levels on OA. This provides valuable insights for OA prevention, diagnosis, and treatment strategies. Future research will expand our study to include a broader spectrum of ethnicities and explore the underlying mechanisms involved.

## 1 Introduction

Insulin-like Growth Factor-1 (IGF-1), a polypeptide chain consisting of 70 amino acids, displays insulin-mimetic properties. It is primarily secreted by the liver, accounting for approximately 75% of its production, with the remaining 25% synthesized by muscular and adipose tissues (Yamashita et al., 1987; Steinman, 2020). In the circulatory system, IGF-1 serves as a negative feedback mechanism for the pituitary gland, influencing the secretion of Growth Hormone (GH). Within the bloodstream, IGF-1 binds to both IGF Binding Proteins (IGFBPs) and Acid Labile Subunit (ALS), extending its serum half-life and determining tissue bioavailability (Ding and Wu, 2018). The Insulin-like Growth Factor Receptor-1 (IGFR-1) is a tyrosine kinase transmembrane receptor with a transmembrane heterotetramer structure. Interaction between IGF-1 and the extracellular domain of IGFR-1 initiates receptor activation and phosphorylation, triggering a cascade of biological responses, including cellular migration, proliferation, differentiation, and motility. This receptor plays a pivotal role in various aspects of both normal and pathological growth and differentiation. Furthermore, it is implicated in the modulation of glucose metabolism, inflammation, and immune responses (Heemskerk et al., 1999; Åberg et al., 2020). IGF-1 exerts a significant influence on bone physiology. Notably, diminished serum levels of IGF-1 can adversely affect linear and radial bone growth, with the latter being a critical factor in bone mechanical strength (Dixit et al., 2021).

Recent studies indicate correlations between blood IGF-1 levels and diverse disease states. Elevated IGF-1 may increase risks associated with coronary artery diseases and Type 2 Diabetes, while also showing connections with various pulmonary conditions (Larsson et al., 2020; Jiang et al., 2023). In orthopedic research, there is a growing interest in the interplay between IGF-1 and bone-joint health. However, the literature presents inconsistent views. While some case-control studies have not established a link between increased IGF-1 levels and heightened osteoarthritis (OA) risk (McAlindon et al., 1993; Hochberg et al., 1994). Recent investigations suggest a potential rise in knee and hip arthritis prevalence (Hartley et al., 2021). Additionally, a study found elevated IGF-1 levels in primary OA patients (Pelsma et al., 2021). The interrelationship between IGF-1 and OA, particularly the confounding effect of body mass index (BMI) as a significant variable in OA, has not been extensively dissected (Claessen et al., 2012). Thus, a research gap exists concerning the specific impact of IGF-1 levels on different types of OA and whether osteoarthritis influences IGF-1 levels.

With the continual increase and public availability of various Genome-Wide Association Studies (GWAS) data through sequencing, Mendelian Randomization (MR) has emerged as a widely adopted research methodology. Due to the inherently random distribution of genetic factors, two-sample MR can simulate the design of a randomized trial without incurring substantial human, material, and time costs. This approach is typically less susceptible to reverse causality and confounding factors (Davey Smith and Hemani, 2014). The aim of this study is to employ the two-sample MR methodology, utilizing its various combinatory forms, to investigate the bidirectional causal relationship between IGF-1 and OA in different body parts. Additionally, we seek to explore the mediating role of BMI in the impact of IGF-1 levels on four types of OA.

## 2 Materials and methods

### 2.1 Study design

**FIGURE 1:**
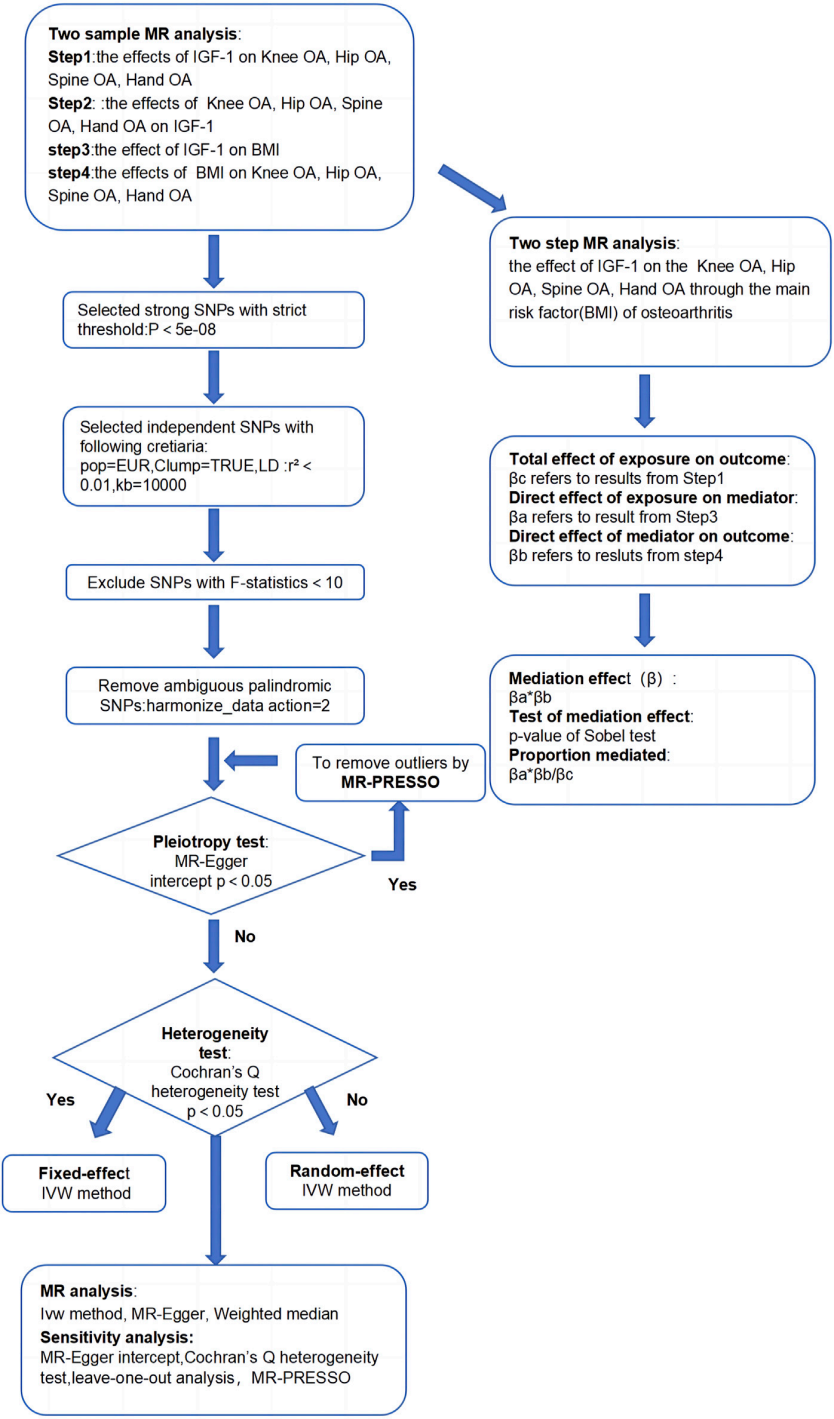
Flowchart of this study.

**FIGURE 2:**
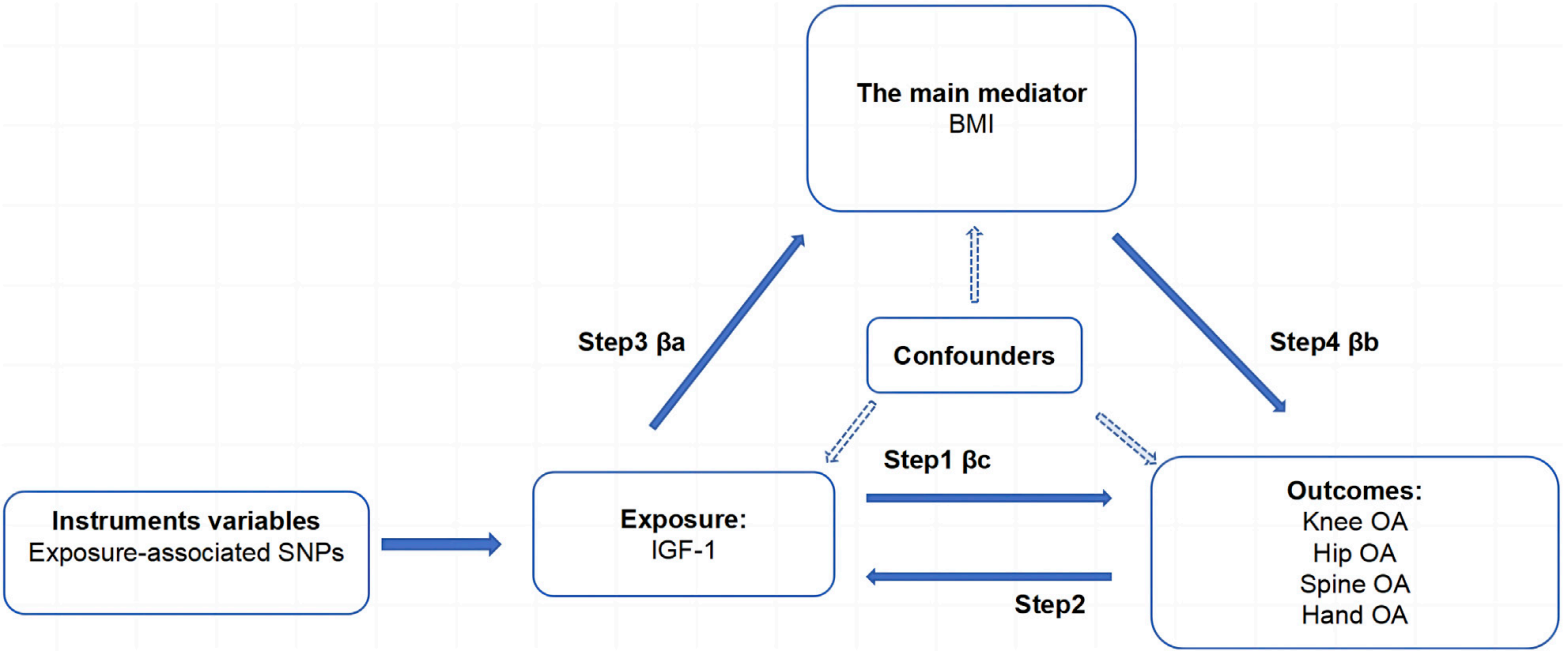
Acyclic graph for our MR analysis: βc, representing the total effect of exposure on the outcome, is determined using the two-sample MR method (i.e., IGF-1 levels as exposure and four types of OA as outcomes); βa and βb represent the indirect effect, where βa is the effect of IGF-1 levels on BMI, and βb is the effect of BMI on OA; The mediation ratio, calculated as βa*βb/βc, represents the proportion of the mediation effect in the total effect

This study aimed to establish the causal relationship between IGF-1 levels and OA in various body parts, along with exploring BMI’s role as a mediator. We utilized two-sample MR analysis and its combination forms to assess bidirectional causality for knee, hip, hand, and spine OA. The effect of IGF-1 on OA via BMI was explored through a two-step MR analysis. Essential assumptions for the two-sample Mendelian analysis included: 1) instrumental factors directly related to exposure; 2) instrumental variables and other confounders are uncorrelated; 3) genetic variation affects outcomes only through exposure (Davies et al., 2018). All our processes are shown in Figures 1, 2.

### 2.2 The data source of IGF-1, OA, and BMI

We obtained GWAS data on IGF-1, OA, and BMI from different studies to minimize sample overlap. The GWAS data of IGF-1 were obtained from the IEU OpenGWAS project (https://gwas.mrcieu.ac.uk/), specifically the study involving whole-exome imputation and fine-mapping analyses (Barton et al., 2021), with the ID number is “ebi-a-GCST90025989”. This dataset comprised of 435,516 samples and 4,231,359 SNPs, with individuals of European ethnicity. The GWAS data of OA were sourced from the musculoskeletal knowledge portal (MSK-KP) (https://msk.hugeamp.org/), representing a meta-analysis of the largest OA GWAS, including up to 826,690 individuals (177,517 of whom were OA patients) (Boer et al., 2021). Four types of OA (knee OA, hip OA, hand OA, and spine OA) were included, with the sample sizes ranging from 303,782 to 396,054 and SNP counts ranging from 15,712,743 to 22,173,239. Approximately 85% of the individuals in this dataset are of European ethnicity. For BMI, a major risk factor for osteoarthritis, were obtained GWAS data from a meta-analysis published by the Genetic Investigation of Anthropometric Traits (GIANT) Consortium. The BMI data, available in the IEU OpenGWAS project under the ID number “ieu-b-40”, contains a sample size of 681,275, a SNP count of 2,336,260, and a population of European ethnicity. Detailed information on the GWAS data used in the study can be found in Supplementary Table S1. The GWAS datasets in our research, sourced from the IEU OpenGWAS project, MSK-KP, and GIANT Consortium, are publicly available and have been granted the necessary ethical approvals. Our study, leveraging these publicly available datasets, did not require separate ethical approval.

### 2.3 Selection of instrumental variables (IVs)

The screening of IVs followed the criteria outlined below: Single-nucleotide polymorphisms (SNPs) associated with exposure at the genome-wide significance level (P ≤ 5 × 10^-8^) were selected. To exclude pairwise linkage disequilibrium (LD), the threshold was set to r^2^ < 0.001, and the genetic distance to the clumping window was set at 10,000 kbp to ensure the independence of SNPs. The European 1000 Genomes Project served as a reference. Harmonizing exposure and outcome data involved setting the action = 2 to remove ambiguous palindromic SNPs, retaining SNPs with MR _Keeper = True. The F-statistic for the SNPs included in each exposure was calculated using the formula: F = r^2^*(N-k-1)/[(1-r^2^)*k]. Here, r^2^ represents the fraction of exposure variance explained by IVs, N is the sample size, and k is the number of IVs. IVs with F ≥ 10 were considered high intensity and retained. Mendelian Randomization Pleiotropy Residual Sum and Outliers (MR-PRESSO) tests were performed to remove outliers when horizontal pleiotropy was tested by egger-intercept with P < 0.05 The remaining SNPs were retained as IVs. When conducting the reverse analysis (IGF-1 as exposure, OA as outcome), the aforementioned criteria were still considered. However, for spine OA, as there were not enough IVs (≥3) available when establishing the P threshold as less than or equal to 5 × 10^–8^, the threshold was adjusted to P ≤ 5 × 10^–6^ based on findings from previous studies (Ference et al., 2015), ensuring an adequate number of IVs (≥3) for Spine OA. Further details of the selected IVs are presented in Table 1.

**TABLE 1:** Genetic instruments selected for exposure.

Exposure	Outcome	SNPs	*R* ^2^	F Statistic	Notes
IGF-1	Knee OA	387	0.102	127.41	NA
IGF-1	Hip OA	388	0.103	128.90	NA
IGF-1	Spine OA	388	0.105	128.73	NA
IGF-1	Hand OA	386	0.103	129.29	MR-PRESSO was used to remove outliers to make sure MR-Egger intercept *P* > 0.05
Knee OA	IGF-1	9	0.00088	36.37–38.88	MR-PRESSO was used to remove outliers to make sure MR-Egger intercept *P* > 0.05
Hip OA	IGF-1	23	0.0029	44.48	NA
Spine OA	IGF-1	7	0.00049	20.81–23.43	The threshold of pval.exposure was set to P < 5e-06 to obtain enough IVs(≥3) to perform Mendelian analysis
Hand OA	IGF-1	3	0.00034	34.68	NA
IGF-1	BMI	305	0.076	118.16	NA
BMI	Knee OA	493	0.048	53.50–82.07	NA
BMI	Hip OA	491	0.048	53.50–82.06	NA
BMI	Spine OA	493	0.048	53.47–82.01	NA
BMI	Hand OA	491	0.048	53.49–82.05	NA

IGF-1, insulin-like growth factor 1; Knee OA, knee osteoarthritis; Hip OA, hip osteoarthritis; Spine OA; spine osteoarthritis; BMI: body mass index; SNPs, single-nucleotide polymorphisms.

### 2.4 MR analysis

To thoroughly assess causal relationships, we utilized three methods: Inverse Variance Weighted (IVW), MR-Egger regression, and Weighted Median (WM). IVW served as the primary method due to its effective estimation, assuming all SNPs are valid instrumental variables (IVs) (Burgess et al., 2013). MR-Egger regression, relying on the instrument strength independent of direct effect (InSIDE) assumption, provides consistent causal estimates if met. However, it has lower power and higher type 1 error compared to IVW (Burgess and Thompson, 2017). The WM method excels in accurately determining causal effects, especially when the InSIDE assumption does not hold. It is more effective than MR-Egger in reducing type I errors (Bowden et al., 2016).

### 2.5 Sensitivity analysis

Sensitivity analyses in our MR study included Egger intercept, MR-PRESSO, heterogeneity analysis, and leave-one-out analysis. During IV selection, Egger intercept and MR-PRESSO were pivotal. The Egger intercept assessed horizontal pleiotropy deviation from zero, indicating a greater probability of pleiotropy (Bowden et al., 2015). MR-PRESSO was then utilized to identify and remove outliers, followed by a reassessment of Egger intercept to confirm the absence of horizontal pleiotropy (*p* > 0.05). During the MR analysis, heterogeneity was assessed using the Cochran Q test to quantify it. In cases of observed heterogeneity among IVs, the multiplicative random effects model of IVW (MRE-IVW) was employed. Otherwise, results from the fixed model of IVW were maintained (Gill, 2020). The leave-one-out analysis evaluated pleiotropy’s impact on causal effects by excluding one SNP at a time and repeating MR analysis. Stable and reliable MR results were confirmed if the estimates aligned with the distribution of the “ALL” line (Burgess et al., 2017). For comprehensive sensitivity analysis results, please refer to Supplementary Table S2.

### 2.6 Bi-direction and two-step MR

We performed a four-step two-sample MR analysis. Step 1: Mendelian analysis of IGF-1 on four types of OA. Step 2: Mendelian analysis of four types of OA on IGF-1. Step 3: Mendelian analysis of IGF-1 on BMI. Step 4: Mendelian analysis of BMI on the four types of OA. The steps 1 and 2 were combined into a bidirectional Mendelian analysis, exploring primary and reverse causal relationships between IGF-1 and the four types of OA. Steps 1, 3, and 4 were combined into a two-step Mendelian analysis to explore the mediating effect of BMI in the relationship between IGF-1 and each type of osteoarthritis. In this analysis, βc represents the total effect, and βa and βb are the direct effects of IGF-1 on BMI and BMI on the four types of OA, respectively. The specific calculation method for the mediating effect is shown in Figures 1, 2.

### 2.7 Statistical analysis

*P*-values obtained after multiple comparisons were adjusted using the Benjamin-Hochberg (BH) method to establish a false discovery rate (FDR) - corrected significance level of q < 0.05 (Benjamini and Hochberg, 1995). To calculate 95% confidence intervals (CI) for the mediating effects, the delta method was utilized (Lynch and Walsh, 1998). The validity of the mediating effect was tested using the Sobel test, suggesting the significance of the mediating effect when *P* < 0.05 (Sobel, 1982). The effect estimate for OAs, treated as a dichotomous variable, was presented as the odds ratio (OR). For the continuous variable IGF-1 and BMI, the effect estimate was presented as beta. Mediation effect results were expressed as Beta, accompanied by a 95% CI. All statistical analyses were conducted using R statistical software with the TwoSampleMR, MRPRESSO, and fdrtool Packages (version 4.3.1, R Foundation for Statistical Computing, Vienna, Austria, 2023; https://www.R-project.org).

## 3 Results

### 3.1 Step 1: IGF-1 levels on four types of OA

**FIGURE 3:**
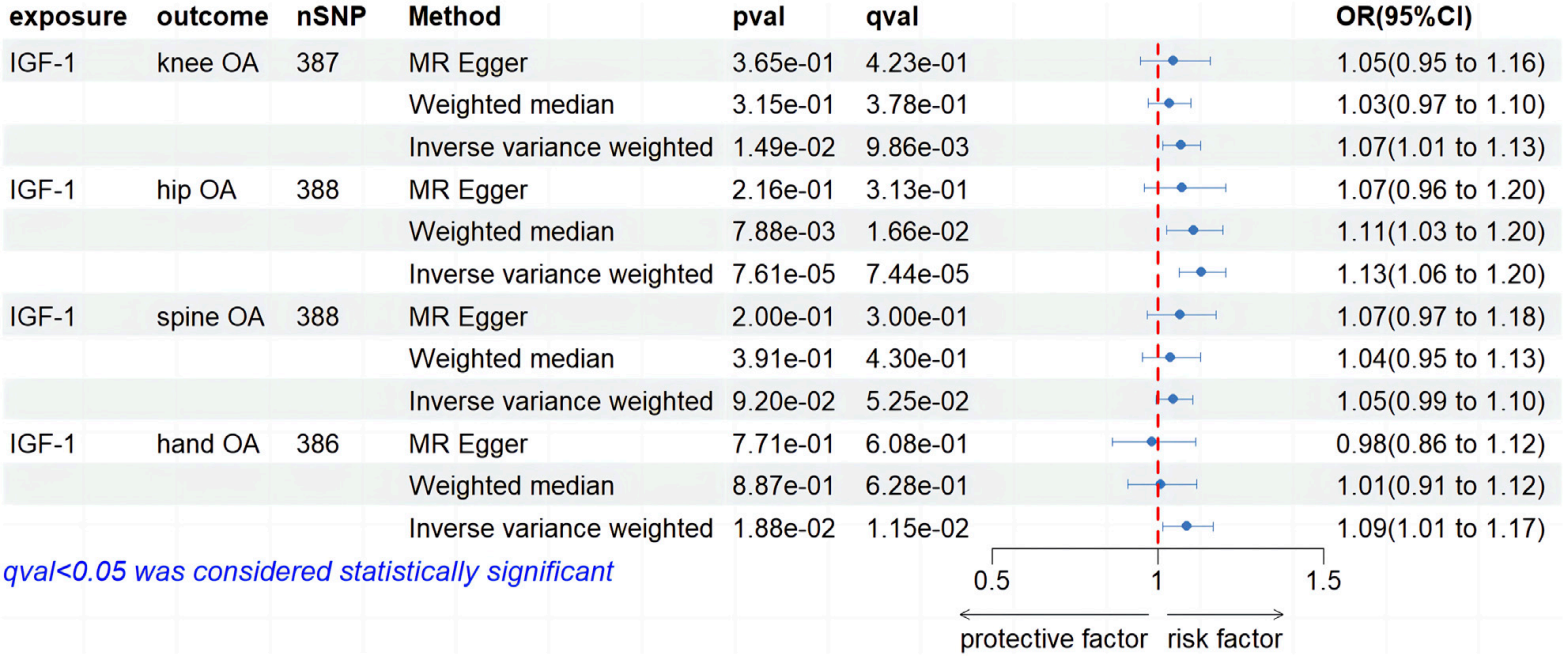
Forest plot of MR results of IGF-1 levels on four types of OA.

As shown in Supplementary Table S1, the outlined process yielded 387,388,388, and 386 SNPs as IVs for conducting MR analysis on knee OA, hip OA, hand OA, and spine OA, respectively, with IGF-1 levels serving as the exposure. The forest plot in Figure 3 reveals the causal effect of IGF-1levels on knee OA. The IVW method, employed as the primary approach, indicated a statistically significant increase in the risk of knee OA associated with IGF-1 (OR, 1.07; 95% CI, 1.01–1.03, *p* = 1.49e-01, q = 9.86e-03). Although the MR-Egger and WM methods did not yield statistically significant results (OR, 1.05; 95% CI, 0.95–1.16, *p* = 3.65e-01, q = 4.23e-01; OR, 1.03; 95% CI, 0.97–1.10, *p* = 3.15e-01, q = 3.78e-01), these findings did not impact the primary outcomes obtained through the IVW method. Regarding the causal effect of IGF-1 levels on hip OA, the IVW results demonstrated a significant increase in the risk of hip OA associated with IGF-1 (OR, 1.13; 95% CI, 1.06–1.20, *p* = 7.61e-05, q = 7.44e-05). The WM method displayed agreement with the findings obtained through the IVW approach (OR. 1.11; 95% CI, 1.03–1.20, *p* = 7.88e-03, q = 1.66e-02), while MR-Egger analysis did not yield statistically significant findings (OR, 1.07; 95% CI, 0.96–1.20, *p* = 2.16e-01, q = 3.13e-01). In the investigation of the causal effect of IGF-1 levels on Spine OA, IVW analysis did not identify a significant increase in the risk of Spine OA associated with IGF-1 (OR, 1.05; 95% CI, 0.99–1.10; *p* = 9.20e-02; q = 5.52e-02). Both MR-Egger and WM analyses demonstrated consistency with IVW results (OR, 1.07; 95% CI, 0.97–1.18, *p* = 2.00e-01, q = 3.00e-01; OR, 1.04; 95% CI, 0.95–1.13, *p* = 3.91e-01, q = 4.30e-01). For the causal relationship between IGF-1 levels and hand OA, the IVW analysis revealed a significant increase in the risk of hand OA associated with IGF-1 levels (OR, 1.09; 95% CI, 1.01–1.17, *p* = 1.88e-02, q = 1.15e-02). However, the MR-Egger and WM analyses did not yield statistically significant results (OR, 0.98; 95% CI, 0.86–1.12, *p* = 7.71e-01, q = 6.08e-01; OR, 1.01; 95% CI, 0.91–1.12, *p* = 8.87e-01, q = 6.28e-01). As shown in Supplementary Table S2, heterogeneity was observed in all of the above results, with no evidence of horizontal pleiotropy. The leave-one-out method was employed to test the stability of the results, and the outcomes are shown in the Supplementary Figures S1A–D.

### 3.2 Step 2: four types of OA on IGF-1 levels

**FIGURE 4:**
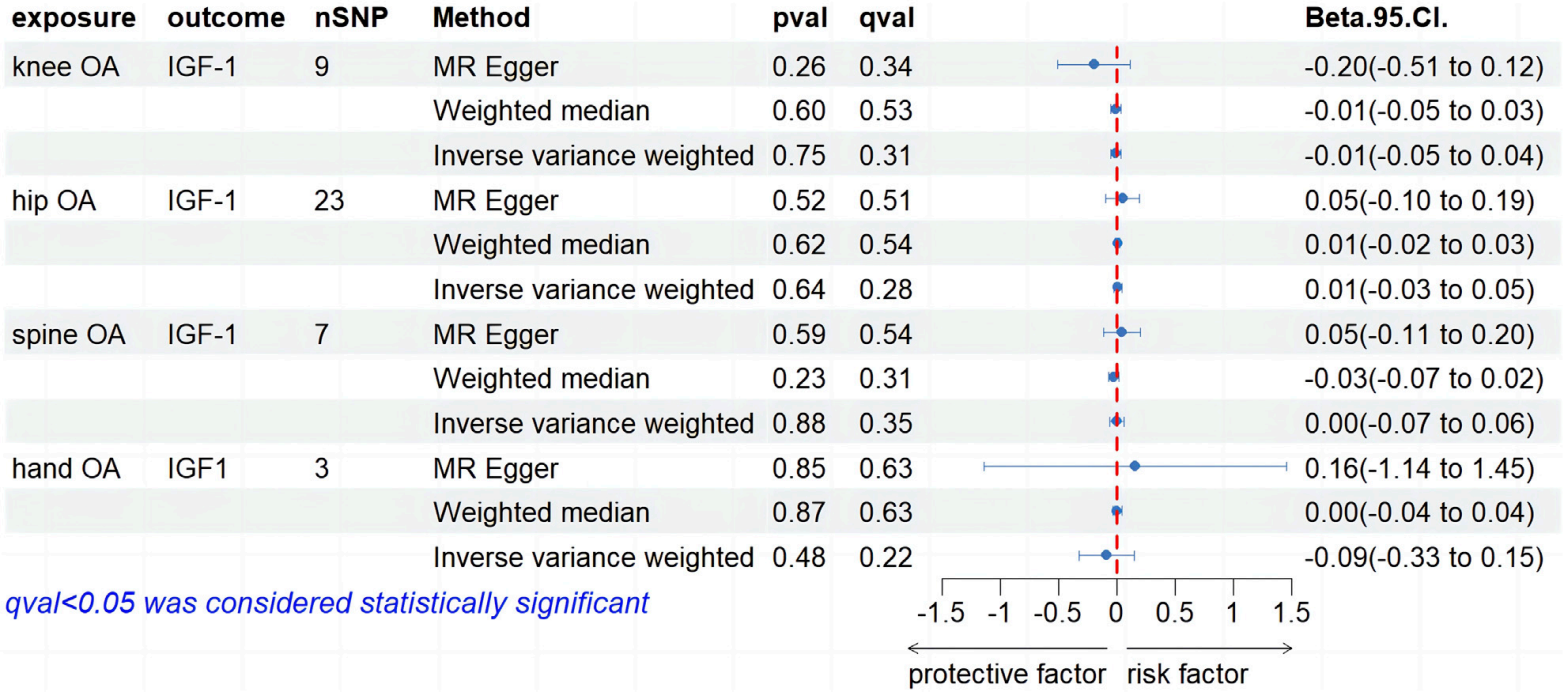
Forest plot of MR results of four types of OA on IGF-1 levels.

Our screening procedures identified and utilized 9, 23, 7, and 3 SNPs as IVs for knee, hip, spine, and hand OA, considering IGF-1 levels as an outcome. All MR outcomes are depicted as forest plots in Figure 4. Regarding the causal impact of knee OA on IGF-1 levels, no statistically significant findings were observed in the IVW result (Beta, −0.01; 95% CI, −0.05–0.04, p = 0.75, q = 0.3). Both the MR-Egger and weighted median (WM) methods exhibited consistency with the IVW method (Beta, −0.20; 95% CI, −0.51–0.12, *p* = 0.26, q = 0.34; Beta, −0.01; 95% CI, −0.05–0.03, *p* = 0.60, q = 0.53). In terms of the causal effect of hip OA on IGF-1, the IVW method did not yield a significant causal relationship (Beta, 0.01; 95% CI, −0.03–0.05, *p* = 0.64, q = 0.28). MR-Egger and WM both failed to find evidence of a causal relationship (Beta. 0.05; 95% CI, −0.10–0.19, *p* = 0.52, q = 0.51; Beta, 0.01; 95% CI, −0.02–0.03, *p* = 0.52, q = 0.51). When examining the causal effect of spine OA on IGF-1, the IVW method revealed that spine OA did not significantly increase IGF-1 levels (Beta, 0.00; 95% CI, −0.07–0.06, *p* = 0.88, q = 0.35). The results from MR-Egger with the WM method were consistent with this finding (Beta, 0.05; 95% CI, −0.11–0.20, *p* = 0.59, q = 0.54; Beta, −0.03; 95% CI, −0.07–0.02, *p* = 0.23, q = 0.31). No significant positive associations between hand OA and IGF-1 were observed using the IVW method. The MR-Egger and WM methods yielded consistent results with the IVW method. As is shown in Supplementary Table S2, heterogeneity was observed in all four results above, and horizontal pleiotropy was not found. Supplementary Figures S1E–H provides detailed leave-one-out results for the aforementioned findings.

### 3.3 Step 3: IGF-1 levels on BMI

**FIGURE 5:**
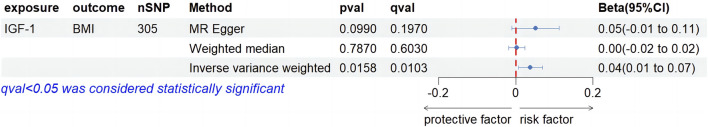
Forest plot of MR results of IGF-1 levels on BMI.

A comprehensive screening process led us to identify and select a total of 305 SNPs as IVs for IGF-1 levels. The MR analysis results, depicted in Figure 5, indicate a positive causal relationship between IGF-1 levels and BMI, as evidenced by the inverse IVW method (Beta, 0.04; 95% CI, 0.01–0.07, *p* = 0.0158, q = 0.0103). However, the MR-Egger and WM methods did not yield any significant findings (Beta. 0.05; 95% CI, −0.01–0.11, *p* = 0.0990, q = 0.1970; Beta,0.00; 95% CI,−0.02–0.02, *p* = 0.7870, q = 0.6030). Nevertheless, these non-significant results did not undermine our interpretation of the primary IVW result regarding causality. As shown in Supplementary Table S2, the heterogeneity test indicated the presence of heterogeneity, while the Egger intercept did not detect any evidence of horizontal pleiotropy. The leave-one-out analysis shown in Supplementary Figure S2A demonstrates the stability of our conclusions.

### 3.4 Step 4: BMI on four types of OA

**FIGURE 6:**
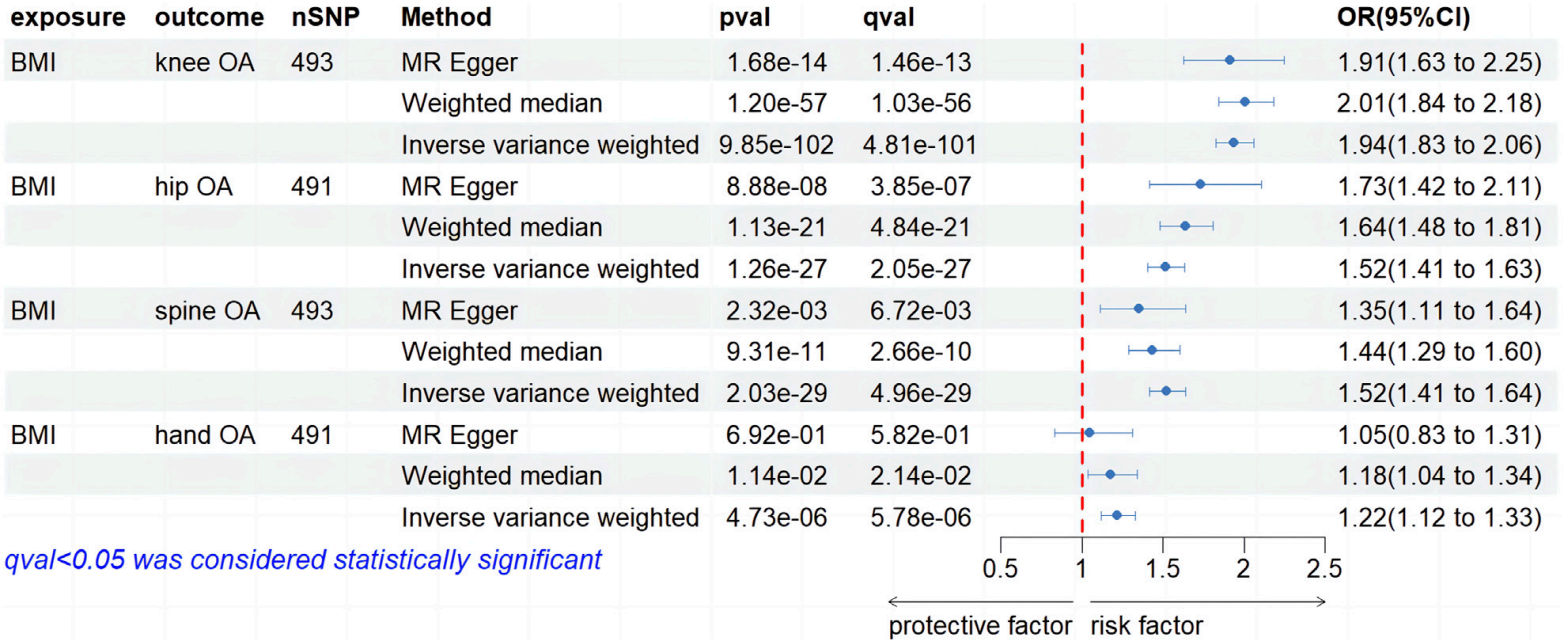
Forest plot of MR results of BMI on four types of OA.

We screened 492, 490, 492, and 490 SNPs as IVs for two-sample MR analysis assessing the impact of BMI on four types of OA. Figure 6 illustrates the causal relationship between BMI and four types of OA. IVW suggested that IGF-1 significantly increased the risk of developing knee OA (OR, 1.94; 95% CI, 1.83–2.06, *p* = 9.85e-102, *q* = 4.81e-101). Results from MR-Egger and WM were consistent with the IVW results (OR, 1.91; 95% CI, 1.63–2.25, *p* = 1.68e-14, *q* = 1.46e-13; OR, 2.01; 95% CI, 1.84–2.18, *p* = 1.20e-57, q = 1.03e-56). Regarding the causality of IGF-1 on hip OA, IVW suggested that enhanced BMI significantly increased the risk of hip OA (OR, 1.52; 95% CI, 1.41–1.63, *p* = 1.26e-27, *q* = 2.05e-27). The MR-Egger and WM results were consistent with the IVW result (OR, 1.73; 95% CI, 1.42–2.11, *p* = 8.88e-08, *q* = 3.85e-07; OR, 1.64; 95% CI, 1.48–1.81, *p* = 1.13e-21, *q* = 4.84e-21). In the investigation of the impact of IGF-1 on spine OA, IVW method demonstrated the statistical significance of the result (OR, 1.52; 95% CI, 1.41–1.63, *p* = 2.03e-29, *q* = 4.96e-29). MR-Egger and WM methods were consistent with the IVW result (OR, 1.35; 95% CI, 1.11–1.64, *p* = 2.32e-03, *q* = 6.76e-03; OR, 1.44; 95% CI, 1.29–1.60, *p* = 9.31e-11, *q* = 2.66e-10). In the causality of BMI on hand OA, IVW method yielded a significant outcome (OR, 1.22; 95% CI, 1.12–1.33, *p* = 4.73e-06, *q* = 5.78e-06), suggesting a positive association between higher BMI and hand OA risk. The WM method corroborated with the result (OR, 1.18; 95% CI, 1.04–1.34, *p* = 1.14e-02, *q* = 2.14e-02), while MR-Egger did not yield statistical significance (OR, 1.05; 95% CI, 0.83–1.31, *p* = 6.92e-01, *q* = 5.82e-01). However, it did not affect our judgment of the conclusion. For detailed sensitivity analyses of the aforementioned four studies, please refer to Supplementary Table S2. The heterogeneity test suggests the presence of heterogeneity in the four results, while the Egger intercept did not find any evidence of horizontal pleiotropy. The findings of the leave-one-out analysis conducted above are depicted in Supplementary Figures S2B–E, providing substantial evidence to support our conclusions.

### 3.5 Bi-direction MR

Based on the findings from Step 1 and Step 2, the bidirectional MR analysis has led to the following conclusions: Elevated levels of IGF-1 increase the susceptibility to knee OA, while knee OA does not exert any influence on IGF-1 levels; Elevated levels of IGF-1 are associated with an increased vulnerability to hip OA. Conversely, hip OA does not have any impact on IGF-1 levels; No causal relationship has been established between the level of IGF-1 and spine OA; The elevated level of IGF-1 amplifies the risk of hand OA, and hand OA does not have any effect on IGF-1 levels.

### 3.6 Two-step MR

Based on the findings of Step 1, Step 3, and Step 4, the mediating role of BMI in the relationship between IGF-1 and four types of OA has been shown in Figure 2. The results obtained have been summarized in Table 2. The mediating effect of BMI in increasing the risk of knee OA through IGF-1 was found to be β (0.025; 95% CI, 0.005–0.046). The mediation proportion is 37.07% (95% CI, 7.41-68.20%). The mediated effect of BMI on the risk of hip OA through IGF-1 was β (0.016; 95% CI, 0.003–0.029). The mediation proportion of 12.86% (95% CI, 2.41–23.31%). While IGF-1 does not directly elevate the risk of spine OA, it can contribute to the development of spine OA by increasing BMI, resulting in a mediated effect of β (0.016; 95% CI, 0.003–0.030). The mediating effect of IGF-1 in increasing the risk of hand OA was β (0.008; 95% CI, 0.001–0.015). The effect is mediated by BMI with a mediation proportion of 9.58% (95% CI, 1.20–17.20%).

**TABLE 2:** Estimated proportion of the effect of IGF-1 levels on four types of OA mediated by BMI.

Exposure	Mediator	Outcome	Total effect:βc (95%CI)	Direct effect:βa (95%CI)	Direct effect:βb (95%CI)	Mediation effect β		Proportion mediated (95%CI)
						Effect size (95%CI)	*P*-value	
IGF-1	BMI	Knee OA	0.067 (0.013, 0.122)	0.038 (0.037, 0.098)	0.663 (0.602, 0.724)	0.025 (0.005, 0.046)	0.016	37.07 (7.41, 68.20)
IGF-1	BMI	Hip OA	0.124 (0.063, 0.186)	0.038 (0.037, 0.098)	0.417 (0.342, 0.491)	0.016 (0.003, 0.029)	0.018	12.86 (2.41, 23.31)
IGF-1	BMI	Spine OA	NA	0.038 (0.037, 0.098)	0.420 (0.347, 0.493)	0.016 (0.003, 0.030)	0.018	NA
IGF-1	BMI	Hand OA	0.084 (0.014, 0.153)	0.038 (0.037, 0.098)	0.1999 (0.114, 0.285)	0.008 (0.001, 0.015)	0.033	9.58 (1.20, 17.20)

IGF-1, insulin-like growth factor 1; Knee OA, knee osteoarthritis; Hip OA, hip osteoarthritis; Spine OA; spine osteoarthritis; BMI: body mass index.

## 4 Discussion

In our study, we utilized the largest-ever GWAS data on OA. Employing the two-sample MR methodology and integrating diverse analytical outcomes, we investigated the relationship between IGF-1 levels and the incidence risk of OA across four different regions of the body. A key focus of our research was to elucidate the mediating role of BMI, recognized as the primary risk factor in OA. Our results suggest that elevated IGF-1 levels in the blood may be associated with an increased risk of knee, hip, and hand OA, without necessarily heighten the risk for spinel OA. Importantly, these four types of OA do not appear to affect IGF-1 levels. In the context of OA risk associated with increased IGF-1 levels, BMI emerges as a pivotal mediator. These findings suggest that while IGF-1 may not directly escalate the risk of spinal OA, it could indirectly influence its development through the promotion of increased BMI.

Our findings demonstrate both agreement and variance compared to existing studies. In a study involving healthy elderly women, an administration of GH leading to increased IGF-1 levels in the blood was significantly correlated with a higher incidence of OA (Blackman et al., 2002), aligning with our results. However, a recent MR study by Hartley et al. explored the relationship between IGF-1 levels and various types of OA, including knee, hip, and hand OA (Hartley et al., 2021), presenting partly different conclusions. They reported that elevated blood IGF-1 levels potentially increased the risk of knee and hip OA but not hand OA. Importantly, they did not address the influence of different OA types on blood IGF-1 levels, which contrasts with our findings. Our study indicates that rising levels of IGF-1 are associated with an increased risk of hand OA. With the utilization of the largest-ever OA GWAS dataset to date, including a larger sample size of participants and more SNPs, our conclusions may show increased significance compared to previous studies. In line with this, research by Lloyd et al. demonstrated that increased serum IGF-1 levels raised the risk of radiographic DIP joint OA (Lloyd et al., 1996), a finding our genetic-level analysis supports. Contrary to Pelsma et al.’s findings of higher IGF-1 levels in OA patients (Pelsma et al., 2021), our study did not observe any impact of the four types of OA on IGF-1 levels. This effect appears to be unidirectional. The literature on IGF-1 and spinal OA is limited; Zhai et al. investigated the association between the 192bp allele of the IGF-1 promoter polymorphism and radiographic OA in the knee, hip, hand, and spine. Their study suggested an increased risk of radiographic OA in the entire cohort, albeit not statistically significant (Zhai et al., 2004). Similarly, our research did not establish a direct causal link between IGF-1 levels and spine OA. This absence of association observed may reflect a genuine phenomenon or could potentially be limited by the current spine OA GWAS data. However, it is worth noting that we have utilized the largest arthritis meta-GWAS dataset to date.

Clinical studies indicating a positive correlation between IGF-1 levels and BMI (Di Giorgio et al., 2014), mirrored in our results and supported by other researchers (Ponzer et al., 1999; Langenberg et al., 2005), stand in contrast to Bredella et al.’s findings of an inverse association (Bredella et al., 2011). Given that Mendelian analyses more closely resemble randomized experiments and can negate reverse causality, we lean toward the validity of our conclusions. Our data, suggesting that BMI increment elevates the risk of developing all four types of arthritis, aligns with the consensus and prior research (Powell et al., 2005). This is attributed to the fact that increased BMI not only adds mechanical stress on the tibiofemoral cartilage but also induces intra-articular inflammation, with compressive stress activating cartilage cell mechanical receptors (He et al., 2021). Similarly, a higher BMI is likely to be positively correlated with chronic inflammation in the body. This inflammation is primarily driven by pro-inflammatory cytokines such as tumor necrosis factor-α (TNFα), interleukin-6 (IL-6), and IL-1β (Monteiro and Azevedo, 2010; Rodríguez-Hernández et al., 2013). Elevated levels of these cytokines have been associated with a decrease in proteoglycan content, resulting in cartilage deformation and reduced stiffness. Consequently, this reduction in proteoglycans may compromise the cartilage’s ability to endure and transmit mechanical stress. Furthermore, a higher prevalence of elevated BMI has been noted in individuals with achondroplasia (Ain et al., 2010; Collins et al., 2018). Elevated BMI is often linked to the development of metabolic syndrome. Meanwhile, IGF-1 plays a multifaced role in various metabolic and inflammatory processes, which can exacerbate OA by intensifying metabolic pathways and inflammatory processes influenced by BMI (Mourkioti and Rosenthal, 2005; Shin and Lee, 2022). This suggests a potential avenue for future research to explore the role of BMI as a pathogenic mechanism in the interaction between IGF-1 and the progression of OA. Our two-step MR analysis further quantifies BMI’s contributory role in the exacerbation of different OA types by IGF-1, showing the highest mediation proportion in the knee joint (37.07%) and the lowest in the hand joint (9.58%). We speculate that this may stem from the differential weight-bearing characteristics of these joints; the knee, as the body’s primary weight-bearing joint, is more susceptible to OA (Guilak, 2011), thus experiencing a greater BMI impact, in contrast to the hand joint, which bears less weight and consequently exhibits a smaller BMI mediation effect.

Although levels of IGF-1 in the blood can increase the risk of OA, either directly or indirectly, the IGF-1 molecule itself has been demonstrated to play a critical role in bone metabolism and the progression of OA, presenting potential for OA treatment. Excessive GH and the resulting increase in serum IGF-1 levels have been linked to heightened bone turnover (Fan et al., 2022). Studies injecting GH into mice, rats, or malignant pituitary growth hormone cell lines (mGH3) producing excessive GH have shown osteoarthritic features, accompanied by increased IGF-1 levels in various areas of the knee joint (Ezzat et al., 1993). Subchondral bone sclerosis, a significant pathological characteristic of advanced OA (Wu et al., 2016), has been associated with the accumulation of IGF-1 and prostaglandin E2 proteins in subchondral bone, inhibiting the expression of Parathyroid Hormone Receptor (PTH-R) in osteoblasts. This resistance to parathyroid hormone affects cyclic Adenosine Monophosphate (cAMP) signaling, leading to sclerosis (Hilal et al., 2001). Moreover, chondrocytes in OA patients secrete more IGF-1 than normal chondrocytes (Doré et al., 1995), and increased local IGF-1 production can amplify the expression of pro-inflammatory cytokines, promoting chondrocyte apoptosis and autophagy, thereby exacerbating OA development. Despite its role in OA progression, IGF-1 also shows potential in OA treatment. Exogenous administration of IGF-1 can upregulate proteoglycan synthesis, stimulate chondrocyte proliferation, enhance cell survival, and downregulate catabolic metabolism induced by chondrocyte proliferation (Wen et al., 2021). Increasing research explores various IGF carrier systems for OA treatment, such as Wu et al.’s study utilizing modRNA transfection techniques to produce engineered adipose-derived mesenchymal stem cells (ADSCs) capable of secreting IGF-1. The application of IGF-1-ADSCs has been effective in slowing OA progression and improving cartilage degradation (Wu et al., 2022). However, these carrier systems are primarily limited to animal studies, and their use in humans raises ethical concerns. It is important to note that our research might contradict some clinical or animal experimental findings, possibly due to their focus on IGF-1 within joint tissues, while we emphasize IGF-1 levels in the blood. For example, Ok et al.’s study demonstrated that localized injections of recombinant human GH increased IGF-1 levels in the synovial fluid but did not affect serum IGF-1 levels (Ok et al., 2020). Therefore, these two scenarios should be considered separately in discussions.

Furthermore, the conclusions drawn from our study may provide valuable insights for developing OA prevention, diagnosis, and treatment strategies. For OA prevention, Monitoring IGF-1 levels in patients’ blood in clinical settings could serve as a predictive tool for assessing the future risk of OA development. However, given the current absence of definitive, large-scale clinical research, we advise caution against manipulating blood IGF-1 levels to reduce this potential risk. This caution is especially relevant considering the crucial role IGF-1 plays in normal physiological growth and development. For example, Laron’s syndrome, an autosomal recessive genetic disorder characterized by GH insensitivity, results in abnormally low IGF-1 levels in the bloodstream. Individuals with this condition typically exhibit near-normal size at birth but experience a pronounced decline in growth velocity thereafter, leading to significantly stunted growth (Rosenbloom et al., 1999; Nashiro et al., 2017). IGF-1 is the sole effective therapeutic intervention for Laron’s syndrome (Latrech et al., 2012). Given the substantial correlation between BMI and OA, strategies aimed at maintaining a healthy weight could be crucial in OA prevention. Interventions that promote physical activity and encourage healthy eating habits, established as longstanding public health objectives for BMI control, may effectively lower OA incidence (Ries and von Tigerstrom, 2010). Regarding OA diagnosis, it is often identified at an advanced stage of disease progression. While various imaging modalities like X-ray, magnetic resonance imaging (MRI), and positron emission tomography (PET) are utilized to aid diagnosis, they are frequently time-consuming and costly (Lawrence et al., 1998; Quinn et al., 2001). Similar to OA prevention, the current research on the diagnostic role of IGF-1 remains limited, and future studies may need to explore this potential avenue. Incorporating genetic risk scores that include variants associated with IGF-1 levels and BMI into clinical practice could be a valuable method to enhance early diagnosis and personalized risk assessment for OA. For OA treatment. Our findings suggest that a more prudent approach would be to focus on interventions targeting BMI, which our data indicate as a concurrent risk factor for all four types of OA. A comprehensive approach involving a combined diet and exercise regimen offers extensive benefits beyond weight reduction, also ameliorating symptoms of osteoarthritis (Messier et al., 2013). Exploration of therapeutic strategies targeting IGF-1 signaling pathways holds promise for treating OA. However, caution should be exercised in pursuing this approach, considering the intricate role of IGF-1 in diverse metabolic and growth processes. Further research is imperative to develop IGF-1 modulators that are safe and efficacious. Currently, we advocate for a holistic approach in managing BMI to effectively mitigate the overall risk of developing osteoarthritis across various body sites and to provide BMI-related therapeutic interventions for OA patients.

Our study stands out with its methodological robustness, novelty, and credibility of data. Employing Mendelian Randomization, it effectively reduces biases due to residual confounders and reverse causation, thereby enhancing the reliability of our findings. This research is pioneering in incorporating spinal osteoarthritis into the investigation of IGF-1 and various types of OA, with BMI serving as a novel mediating variable. This inclusion contributes to a broader understanding of the field. Furthermore, the use of large-sample datasets from relevant databases significantly bolsters the persuasiveness and credibility of our conclusions. Conversely, the study faces limitations related to data granularity and the complexity of instrumental variables. The current lack of detailed stratification in arthritis data, particularly regarding severity, restricts the exploration of the IGF-1 and OA relationship. While the utilization of numerous instrumental variables for IGF-1 and BMI is supported by robust F-statistics and sensitivity tests, it introduces potential challenges due to the inclusion of ineffective IVs. Notably, the GWAS data for IGF-1 and BMI come from European populations, with approximately 85% of the arthritis GWAS data originating from individuals of European ethnicities. Given the genetic disparities and varying living conditions among racial groups, these findings may have greater relevance to European populations. To broaden the scope of this study and ensure its applicability to a wider range of populations, further research incorporating GWAS data specific to diverse ethnicities is essential. The decision to relax the *P*-value threshold for Spine OA IVs in reverse causation analysis, although methodologically sound, suggests a need for a cautious interpretation of this finding. Furthermore, despite utilizing the largest available arthritis GWAS dataset, the sample size and number of SNPs for Spine OA remain limited. The absence of a definitive causal relationship between IGF-1 and Spine OA might be authentic. However, our study revealed a previously unrecognized causal relationship between IGF-1 and hand OA through analysis of larger GWAS data. Expanding research efforts with expanded GWAS datasets could provide further insights into the causal relationship between IGF-1 and Spine OA. Future research will delve into the pathological pathways and mechanisms that connect IGF-1, BMI, and arthritis, as well as the determination of the quantitative ranges for IGF-1 levels and BMI concerning these diseases.

## 5 Conclusion

This MR study demonstrates that elevated levels of IGF-1 may increase the susceptibility to knee, hip, and hand OA. However, there is currently no evidence of an increased risk for spine OA. Interestingly, IGF-1 could amplify the risk of all four types of OA through its impact on BMI. These findings offer valuable insights into the prevention, diagnosis, and treatment strategies for preventing OA. Future research will broaden our study to include a more diverse range of ethnic groups, allowing for a comprehensive exploration of the underlying mechanisms involved.

## Data Availability

The original contributions presented in the study are included in the article/Supplementary Material, further inquiries can be directed to the corresponding authors.

## Abbreviations

ALS, Acid Labile Subunit; ADSCs, adipose-derived mesenchymal stem cells; BH, Benjamin-Hochberg; BMI, body mass index; cAMP, cyclic Adenosine Monophosphate; CI, confidence interval; FDR, false discovery rate; GIANT, Genetic Investigation of Anthropometric Traits; GWAS, Genome-Wide Association Studies; GH, Growth Hormone; IGFBPs, IGF Binding Proteins; IGFR-1, Insulin-like Growth Factor Receptor-1; IGF-1, Insulin-like Growth Factor-1; IL-6, interleukin–6; IVW, Inverse variance weighted; IVs, instrumental variables; MR, Mendelian Randomization; MR-PRESSO, Mendelian randomization pleiotropy residual sum and outliers; MSK-KP, musculoskeletal knowledge portal; MRE-IVW, multiplicative random effects model of IVW; MRI, magnetic resonance imaging; OR, odds ratio; OA, Osteoarthritis; PTH-R, Parathyroid Hormone Receptor; PET, positron emission tomography; SNPs, ingle-nucleotide polymorphisms; TNF-α, stumor necrosis factor-α; WM, weighted median.
