# Supplementary material for: The association between IGF-1 levels and four types of osteoarthritis: a bidirectional and two-step mendelian randomization study
Source: Front Genet. 2024 Jul 10;15:1366138. doi: 10.3389/fgene.2024.1366138 (PMC11266086; doi:10.3389/fgene.2024.1366138)
Supplement: Supplementary file 1 [file Table1.DOCX]

**Supporting information**

**The Association Between IGF-1 Levels and four types of osteoarthritis: A Bidirectional and two-step Mendelian Randomization Study**

**Supplementary table 1.** Detailed GWAS information of IGF-1levels, BMI, and OA on used studies

**Supplementary table 2.** Sensitivity analyses of two-sample Mendelian analysis

**Supplementary Figure 1.** Leave-one-out analysis of bidirectional Mendelian analysis

**Supplementary Figure 2.** Leave-one-out analysis of two-step Mendelian analysis

**Supplementary table 1**. Detailed information on used studies

IGF-1, insulin-like growth factor 1; Knee OA, knee osteoarthritis; Hip OA, hip osteoarthritis; Spine OA; spine osteoarthritis; BMI:body mass index

SNPs, single-nucleotide polymorphism

| **Trait** | Consortium or cohort study | Samplesize | Number of SNPs |  | PubMed ID or web source |
| --- | --- | --- | --- | --- | --- |
| IGF-1 | UK Biobank | 435,516 | 4,231,359 |  | 34226706 |
| Knee OA | Musculoskeletal Knowledge Portal | 396,054 | 22,173,239 |  | https://msk.hugeamp.org/ |
| Hip OA | Musculoskeletal Knowledge Portal | 353,388 | 18,871,781 |  |  |
| Spine OA | Musculoskeletal Knowledge Portal | 333,950 | 19,360,900 |  |  |
| Hand OA | Musculoskeletal Knowledge Portal | 303,782 | 15,712,743 |  |  |
| BMI | Giant | 681,275 | 2,336,260 |  | 30124842 |

**Supplementary table 2.** Sensitivity analyses of two-sample Mendelian analysis

| **Exposure** | **Outcome** | **MR-Egger intercept** | **P of MR-Egger intercept*** | **Cochran’s Q-statistic** | **DF** | **P of Cochran's Q heterogeneity test**† |
| --- | --- | --- | --- | --- | --- | --- |
| IGF-1 | Knee OA | 0.00066 | 0.61 | 1023.57 | 386 | <0.001 |
| IGF-1 | Hip OA | 0.0016 | 0.28 | 835.50 | 387 | <0.001 |
| IGF-1 | Spine OA | -0.00057 | 0.66 | 501.85 | 387 | <0.001 |
| IGF-1 | Hand OA | 0.0032 | 0.06 | 573.29 | 385 | <0.001 |
| Knee OA | IGF-1 | 0.010 | 0.27 | 19.48 | 8 | <0.001 |
| Hip OA | IGF-1 | -0.0029 | 0.58 | 175.05 | 22 | <0.001 |
| Spine OA | IGF-1 | -0.0041 | 0.52 | 27.90 | 6 | <0.001 |
| Hand OA | IGF-1 | -0.022 | 0.77 | 122.07 | 2 | <0.001 |
| IGF-1 | BMI | -0.00038 | 0.62 | 3504.88 | 304 | <0.001 |
| BMI | Knee OA | 0.00026 | 0.85 | 957.27 | 492 | <0.001 |
| BMI | Hip OA | -0.0023 | 0.16 | 894.72 | 490 | <0.001 |
| BMI | Spine OA | 0.0021 | 0.20 | 690.50 | 492 | <0.001 |
| BMI | Hand OA | 0.0027 | 0.15 | 639.30 | 490 | <0.001 |

IGF-1, insulin-like growth factor 1; Knee OA, knee osteoarthritis; Hip OA, hip osteoarthritis; Spine OA, spine osteoarthritis; BMI,body mass index;SNPs, single-nucleotide polymorphisms; *,When there was significant pleiotropy (MR-Egger Intercept test P < 0.05), the MR-PRESSO was used to remove outliers;†,When there was significant heterogeneity (P < 0.05), the random-effect IVW model was used, otherwise the fixed-effect IVW model was used.

**Supplementary figure 1.** Leave-one-out analysis of bidirectional Mendelian analysis


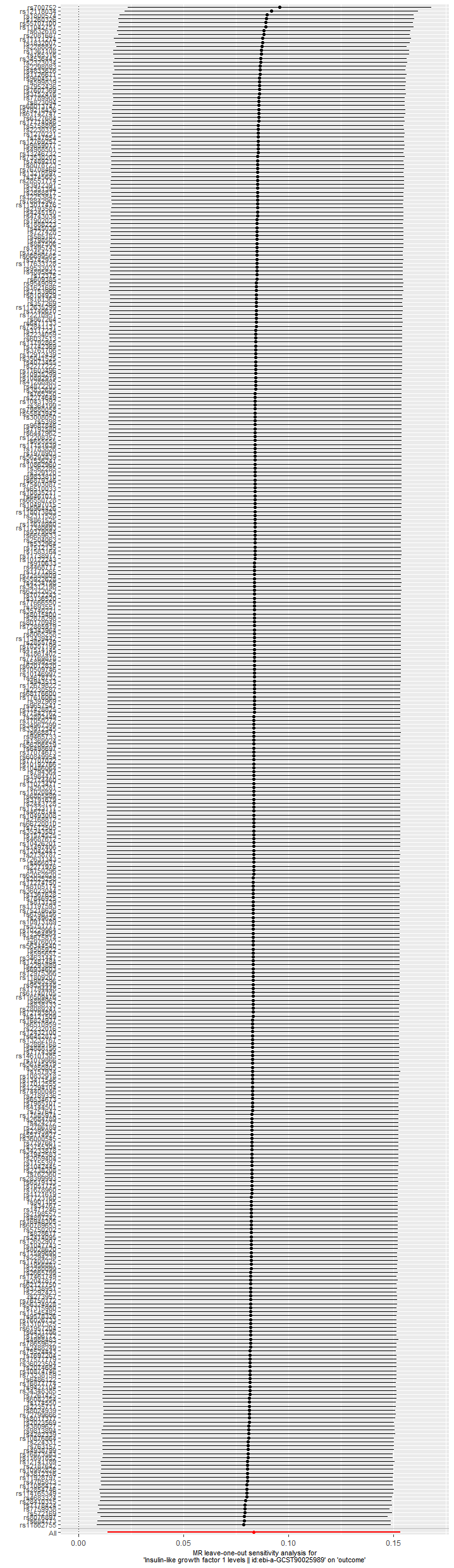

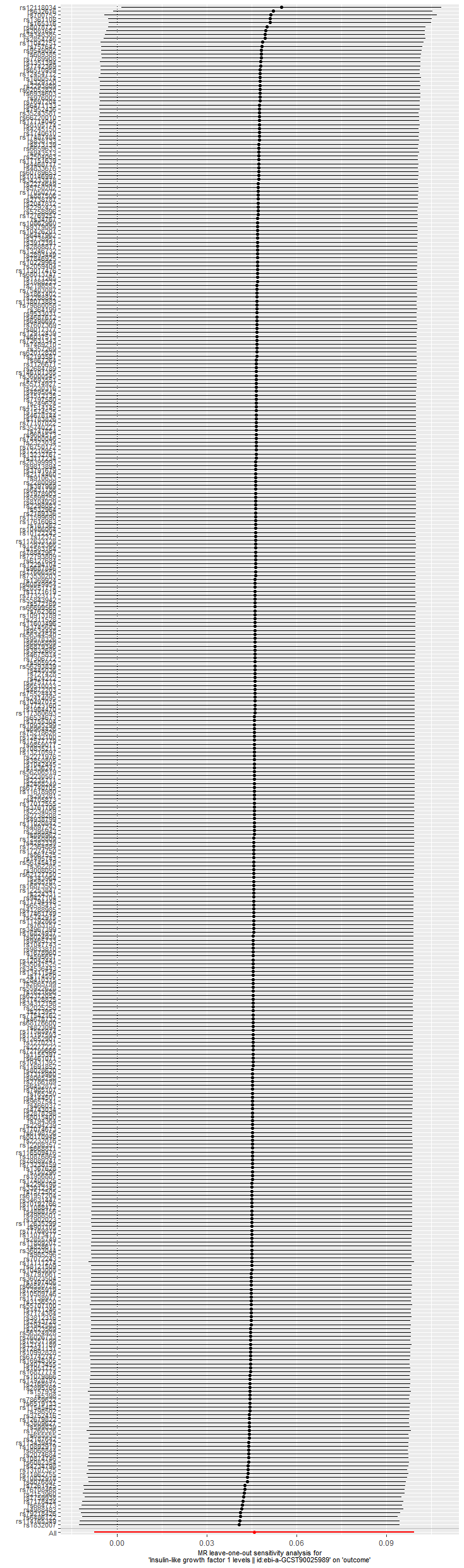

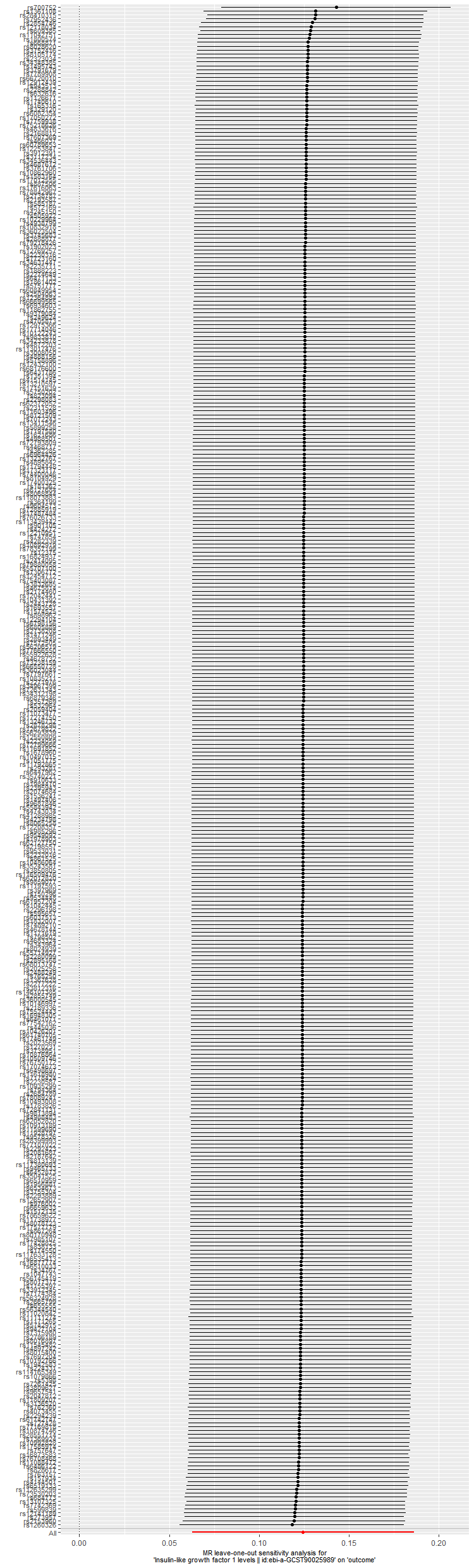

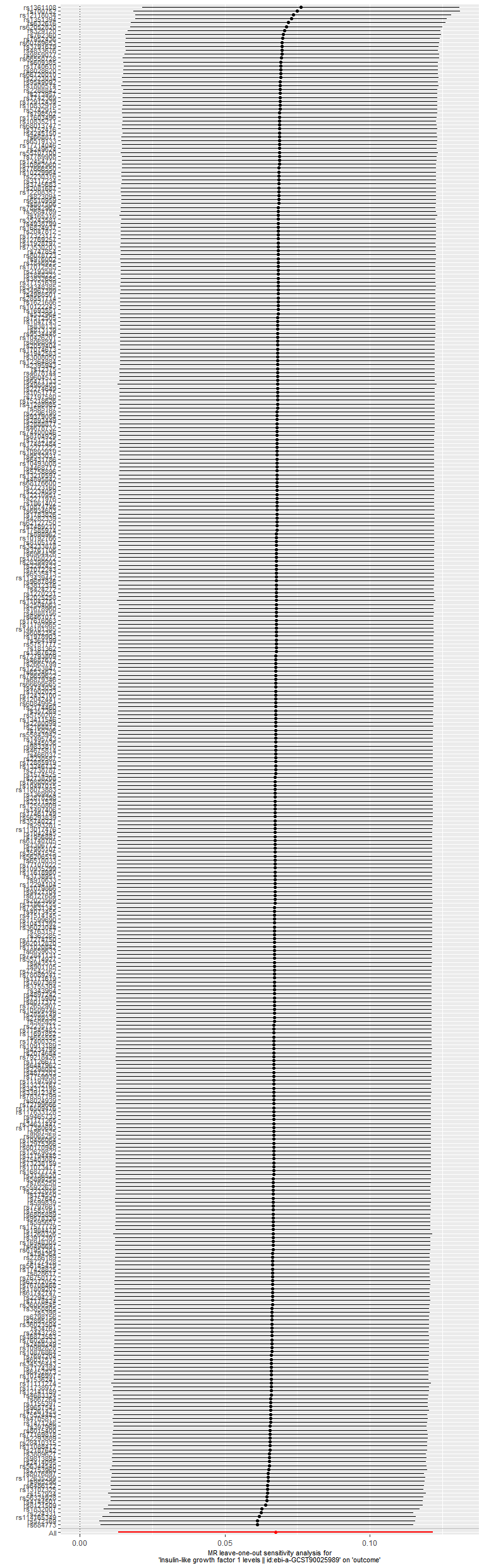
A B C D


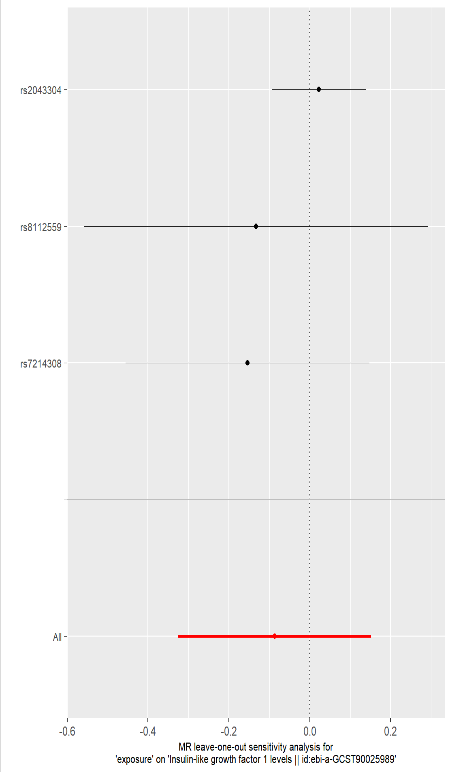

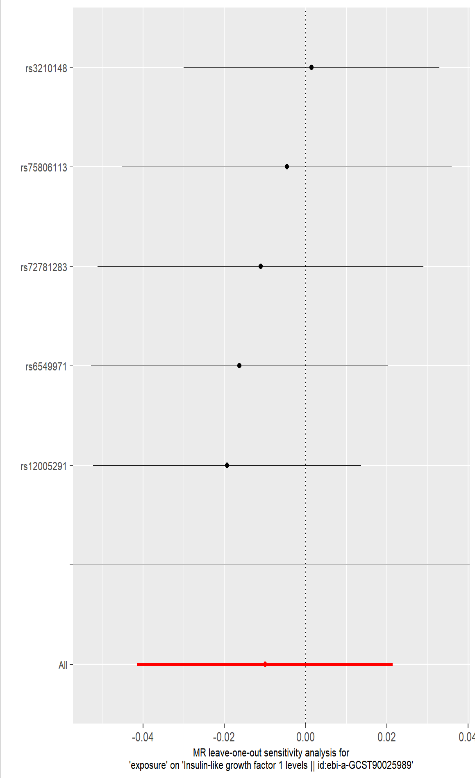

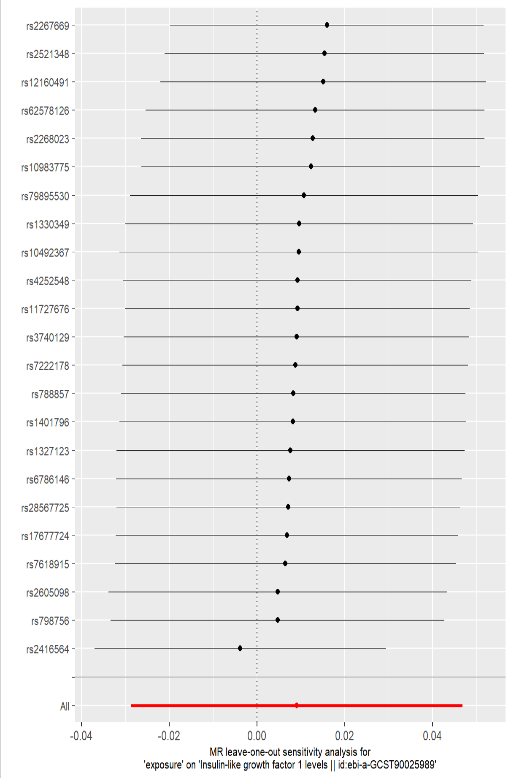

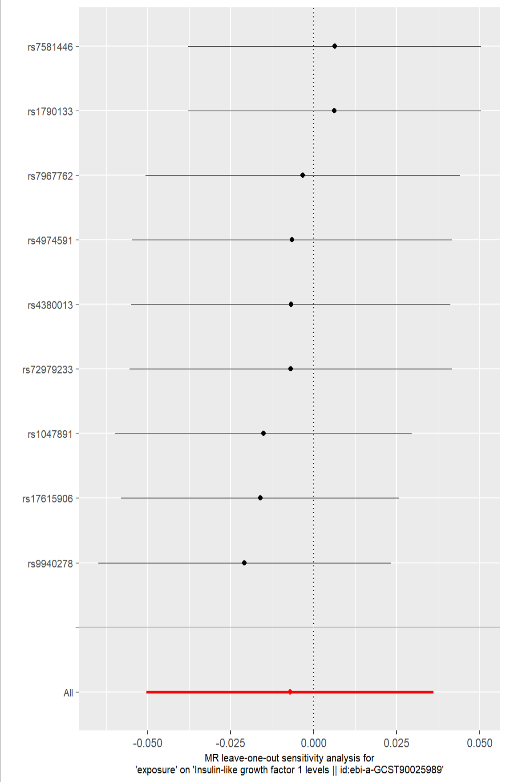


E F G H

A, leave-one-out sensitivity analysis of IGF-1 on Knee OA；B, leave-one-out sensitivity analysis of IGF-1 on Hip OA ;C, leave-one-out sensitivity analysis of IGF-1 on Spine OA;D, leave-one-out sensitivity analysis of IGF-1 on Hand OA;E, leave-one-out sensitivity analysis of Knee OA on IGF-1;F, leave-one-out sensitivity analysis of Hip OA on IGF-1;G, leave-one-out sensitivity analysis of Spine OA on IGF-1,H leave-one-out sensitivity analysis of Hand OA on IGF-1

**Supplementary figure 2.** Leave-one-out analysis of two-step Mendelian analysis


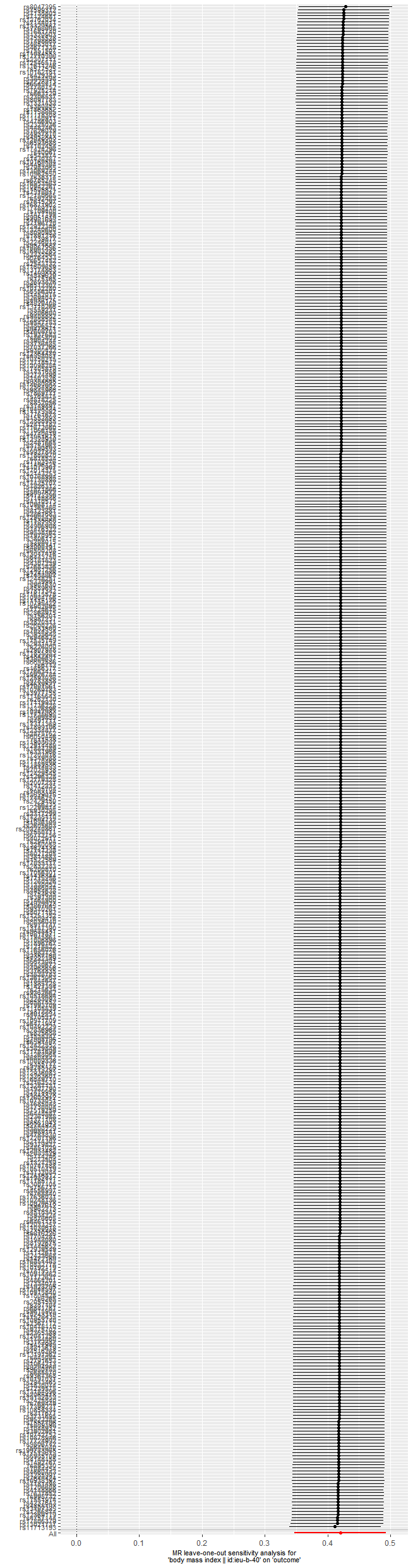

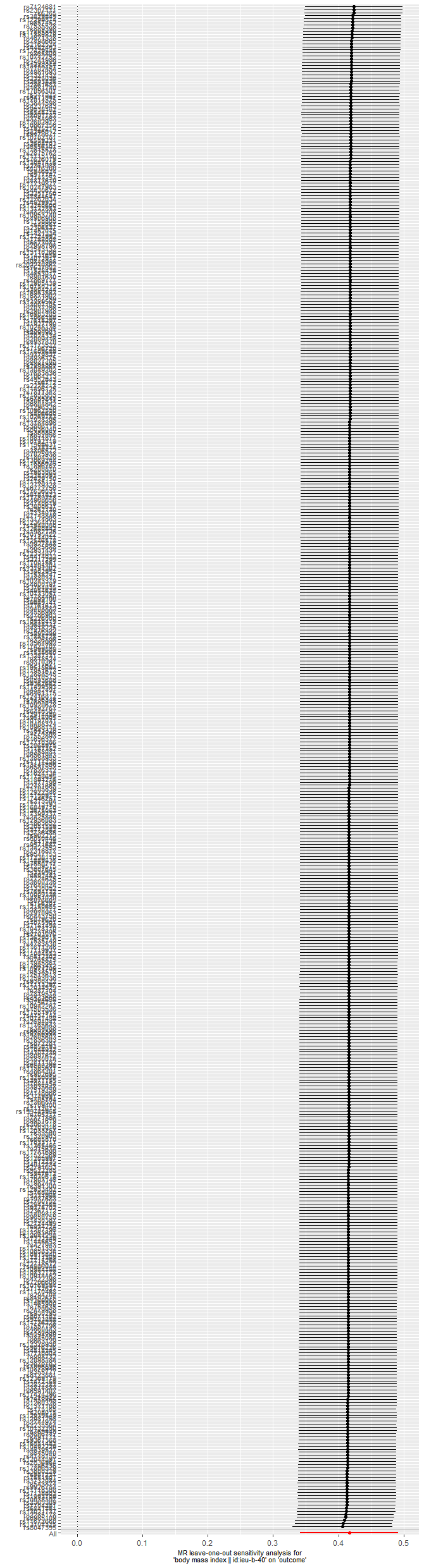

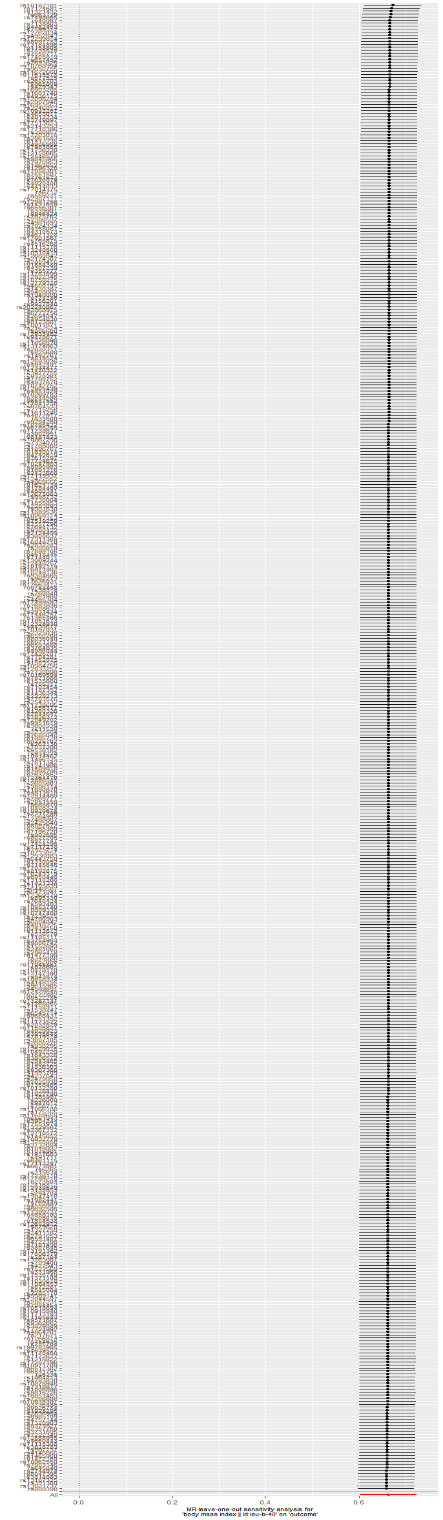

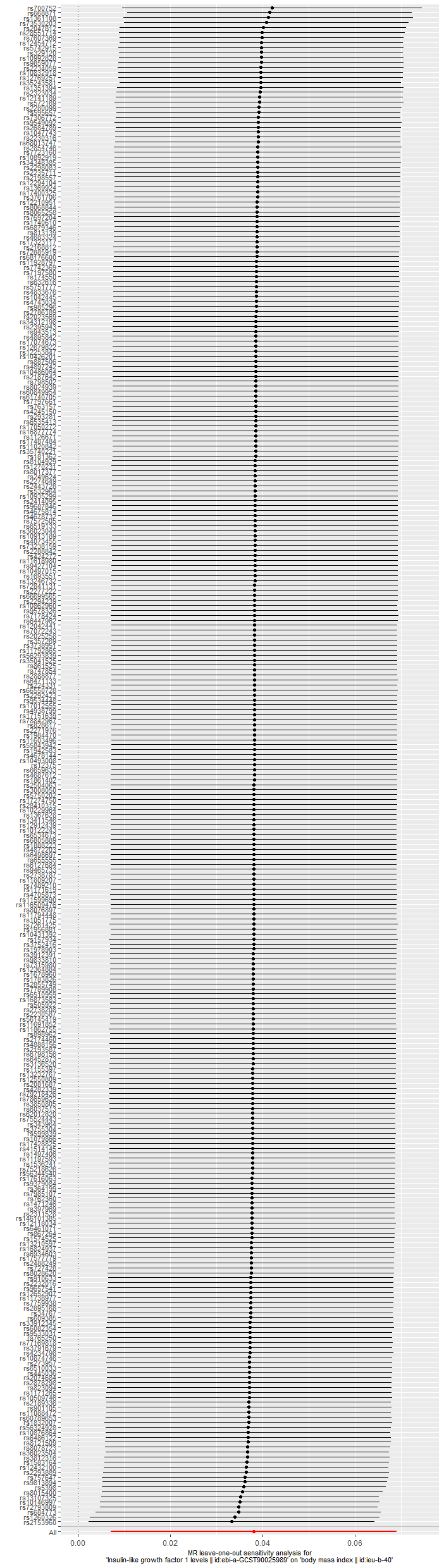


A B C D


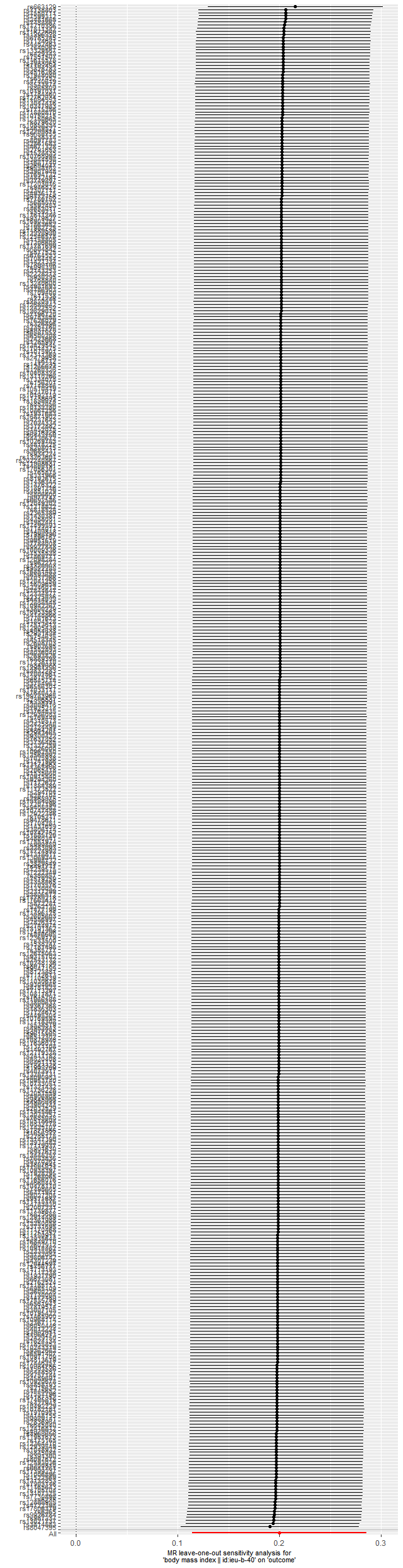


E

A,leave-one-out sensitivity analysis of IGF-1 on BMI；B, leave-one-out sensitivity analysis of IGF-1 on Knee OA ;C, leave-one-out sensitivity analysis of IGF-1 on Hip OA;D, leave-one-out sensitivity analysis of IGF-1 on Spine OA;E, leave-one-out sensitivity analysis of IGF-1 on Hand OA
